# Molecular mechanism of the schedule-dependent synergistic interaction in EGFR-mutant non-small cell lung cancer cell lines treated with paclitaxel and gefitinib

**DOI:** 10.1186/1756-8722-4-5

**Published:** 2011-01-21

**Authors:** Hua Cheng, She-Juan An, Song Dong, Yi-Fang Zhang, Xu-Chao Zhang, Zhi-Hong Chen, Jian-Su, Yi-Long Wu

**Affiliations:** 1 Guangdong Lung Cancer Institute; Medical Research Center of Guangdong General Hospital & Guangdong Academy of Medical Sciences. No.106, Zhongshan 2nd Rd, Guangzhou, Postal code:510080, People's Republic of China; 2 Thoracic oncology, the fifth affiliated hospital of Sun Yat-Sen university. No. 52, Meihua Dong Rd, Zhuhai, Postal code:519000, People's Republic of China

## Abstract

**Background:**

Chemotherapy combined concurrently with TKIs produced a negative interaction and failed to improve survival when compared with chemotherapy or TKIs alone in the treatment of non-small cell lung cancer (NSCLC). The present study investigated the sequence-dependent interaction between paclitaxel and gefitinib and clarified the underlying mechanism.

**Methods:**

The effects on cell proliferation, EGFR signaling pathway, and TGFα expression were evaluated in a panel of human NSCLC cell lines harboring EGFR mutations with three different combination sequences: sequential treatment with paclitaxel followed by gefitinib (T→G), sequential treatment with gefitinib followed by paclitaxel (G→T), or concomitant treatment (T + G).

**Results:**

The sequence-dependent anti-proliferative effects differed between EGFR-TKI-sensitive and -resistant cell lines carrying *EGFR *mutations. A synergistic anti-proliferative activity was obtained with paclitaxel treatment followed by gefitinib in all cell lines, with mean CI values of 0.63 in Hcc827, 0.54 in PC-9, 0.81 in PC-9/GR, and 0.77 in H1650 cells for the T→G sequence. The mean CI values for the G→T sequence were 1.29 in Hcc827, 1.16 in PC-9, 1.52 in PC-9/GR, and 1.5 in H1650 cells. The mean CI values for T+G concomitant treatment were 0.88 in Hcc827, 0.91 in PC-9, 1.05 in PC-9/GR, and 1.18 in H1650 cells. Paclitaxel produced a dose-dependent increase in EGFR phosphorylation. Paclitaxel significantly increased EGFR phosphorylation compared with that in untreated controls (mean differences: +50% in Hcc827, + 56% in PC-9, + 39% in PC-9/GR, and + 69% in H1650 cells; *p *< 0.05). The T→G sequence produced significantly greater inhibition of EGFR phosphorylation compared with the opposite sequence (mean differences: -58% in Hcc827, -38% in PC-9, -35% in PC-9/GR, and -30% in H1650 cells; *p *< 0.05). Addition of a neutralizing anti-TGFα antibody abolished paclitaxel-induced activation of the EGFR pathway in PC-9 and H1650 cells. Sequence-dependent TGFα expression and release are responsible for the sequence-dependent EGFR pathway modulation.

**Conclusion:**

The data suggest that the sequence of paclitaxel followed by gefitinib is an appropriate treatment combination for NSCLC cell lines harboring EGFR mutations. Our results provide molecular evidence to support clinical treatment strategies for patients with lung cancer.

## Background

Despite recent advances in early diagnosis and treatment, non-small cell lung cancer (NSCLC) is still a disease with a poor prognosis. Platinum-based doublet chemotherapy is the mainstay of treatment for advanced NSCLC with good performance status [1,2]. Current data suggest that NSCLC chemotherapy has reached a therapeutic plateau [3,4].

Gefitinib and erlotinib are orally active, reversible Her-1/epidermal growth factor receptor tyrosine kinase inhibitors (EGFR-TKIs). In 2004, researchers found that EGFR-activating mutations correlated with clinical responses [5-7]. The Iressa Pan-Asia Study (IPASS) trial indicated that gefitinib was superior to carboplatin plus paclitaxel as an initial treatment for patients with advanced NSCLC harboring an EGFR mutation [8]. The finding was further supported by two randomized studies (the WJTOG3405 and NEJ 002 trials) that consistently reported a high tumor response rate and progression-free survival (PFS) in patients with an EGFR mutation [9,10]. The EGFR mutation rate was higher in Asian than in western patients, explaining the higher response rate in East Asian patients [11]. Based on these studies, an EGFR mutation is currently the only established predictive factor for EGFR-TKIs.

An increasingly interesting area of clinical research is the development of rationale combinations of cytotoxic drugs with molecularly targeted therapies to increase the therapeutic potential by blocking cancer cell survival mechanisms. Recently, we have shown that the sequence of paclitaxel followed by gefitinib improves the antiproliferative effect compared with other sequences and produced a synergistic effect. We also found the sequence-dependent modulation of EGFR phosphorylation plays a role in this sequence-dependent antiproliferative effect [12]. However, we did not focus on cell lines with mutant EGFR and the exact mechanism underlying the modulation of EGFR phosphorylation remains to be determined. While other studies indicated that TGFα release is responsible for EGFR activation induced by radiotherapy [13,14], we hypothesized that TGFα might play a role in the sequence-dependent antiproliferative effect.

Thus, the present study was performed in NSCLC cell lines harboring EGFR-activating mutations to investigate the synergistic interaction between paclitaxel and gefitinib, and to determine the underlying mechanism(s). We found that sequence-dependent TGFα expression and release were responsible for the sequence-dependent EGFR pathway modulation and sequence-dependent antiproliferative effect.

## Materials and methods

### Drugs and chemicals

Pure gefitinib, kindly provided by AstraZeneca (London, UK), was dissolved in dimethyl sulfoxide (DMSO) as a 20 mM stock solution. Paclitaxel was purchased from Sigma (St. Louis, MO, USA) and was dissolved in DMSO as a 1 mM stock solution. Both drugs were diluted with culture medium before use.

Primary antibodies: anti-pY1068 EGFR (phosphotyrosine-specific EGFR antibody) and anti-β-actin were purchased from Cell Signaling Technology (Danvers, MA, USA), anti-EGFR was purchased from Santa Cruz Biotechnology (Santa Cruz, CA, USA), and anti-TGFα antibody[189-2130.1] was purchased from Abcam (Cambridge, MA, USA).

### Cell lines

The human lung adenocarcinoma cell lines PC-9, Hcc827, and H1650 were kindly provided by Dr. Tony Mok (Chinese University of Hong Kong). These cell lines have been extensively characterized. PC-9 is derived from a patient with adenocarcinoma, harboring an EGFR exon 19 in-frame deletion[E746-A750] that is highly sensitive to EGFR-TKIs[15], Hcc827 is derived from lung adenocarcinomas harboring an EGFR exon 19 in-frame deletion that is highly sensitive to EGFR-TKIs[16]. H1650 carries an EGFR exon 19 in-frame deletion mutation and is resistant to EGFR-TKIs. Martin et al. reported the homozygous deletion of PTEN in H1650 cells, which activated EGFR and contributed to erlotinib resistance[17]. PC-9/GR is a gefitinib-acquired resistant cell line that was established by chronic exposure of PC-9 cells to medium with increasing concentrations of gefitinib. Briefly, PC-9 cells were exposed to 10 nmol/L of gefitinib in medium containing 10% fetal bovine serum, and the concentration was increased in a stepwised manner. Cells that were able to grow in 1 μmol/L gefitinib were obtained 6 month after initial expose. The decreased sensitivity did not recover even the cells were kept in culture for > 4 month without gefitinib. These cells were grown in RPMI 1640 medium supplemented with 10% fetal bovine serum (FBS), penicillin (100 UI/ml), and streptomycin (100 μg/ml) at 37°C in a humidified atmosphere with 5% CO_2 _and harvested with trypsin-EDTA when the cells were in exponential growth.

### Sequencing of EGFR gene

The exons encoding the intracellular domain of EGFR were amplified from genomic DNA and directly sequenced. To further characterize additional mutation, we amplified the EGFR exon 19 to 20 from PC-9 or PC-9/GR cDNA, and the PCR products were subcloned into plasmid vector, then inserts were isolated and sequenced.

### Treatment regimens

Cell viability was determined using the MTT (3-(4,5-dimethylthiazol-2-yl)-2,5- diphenyltetrazolium bromide) assay, according to the method of Mosmann and Carmichael [18,19]. To evaluate the antiproliferative effects of combined treatment, cells were treated with three different sequences: I, pretreated with paclitaxel for 24 h, aspirated and washed once with phosphate-buffered saline (PBS), followed by gefitinib for 48 h; II, pretreated with gefitinib for 48 h, aspirated and washed once with PBS, followed by paclitaxel for 24 h; III, treated concomitantly with paclitaxel plus gefitinib for 48 h, and then incubated in drug-free medium for 24 h. The different drug doses were combined using constant ratios of the IC_50 _values, calculated from previous cytotoxicity tests. Thus, we used 0.125, 0.25, 0.5, 1, 2, and 4 times the IC50 dose in paclitaxel and gefitinib combination doses to calculate the CI value. The results of sequential treatment with paclitaxel and gefitinib were analyzed according to the method of Chou and Talaly [20]. The resulting combination index (CI) represented a quantitative measure of the degree of interaction between different drugs, with CI > 1.1, CI = 0.9-1.1, and CI < 0.9 indicating antagonistic, additive, and synergistic effects, respectively. The CI value was calculated using the CompuSyn software (ComboSyn, Inc., Paramus, NJ, USA).

### Western blot analysis

Cells were seeded at 1 × 10^5 ^per plate on 10-cm plates and left to settle overnight. Cells were then treated by adding fresh medium containing drugs for the desired times. Soluble protein was extracted with the addition of RIPA lysis solution. Lysates were cleared by centrifugation (12000 rpm, 4°C, 15 min), and soluble protein extracts were stored frozen at -80°C. Protein concentrations were quantitated using the bicinchoninic acid assay[21]. Total cell extracts (30 μg of protein/well) were resolved on 8-10% acrylamide Tris-acetate gels, and then proteins were transferred to a polyvinylidene fluoride (PVDF) membrane, blocked for 1 h at room temperature in 5% w/v non-fat milk diluted in Tris-buffered saline Tween 20 (TBST), and finally incubated with appropriate primary antibodies under the conditions recommended by the manufacturers. The blots were then washed with TBST for 30 min and incubated with horseradish peroxidase-conjugated secondary antibody at room temperature for 1 h. Antibody binding was detected using an enhanced chemiluminescence system(Santa Cruz, CA, USA).

To quantify protein levels, films were scanned and analyzed with the Labworks software(World BioHazTec Corp, NM, USA). The relative protein levels were counted using a comparison with the untreated control.

### Real-time polymerase chain reaction (PCR)

To compare the possible influence of TGFα gene expression on the sequence-dependent synergistic effect between paclitaxel and gefitinib, we used PC-9 cells to analyze the sequential modulation of TGFα expression. The primer and probe design, total RNA isolation, cDNA synthesis, and quantification standards for real-time PCR were performed as described previously [22,23]. Total RNA was isolated using the Trizol reagent (Invitrogen) according to the manufacturer's protocol. The integrity of the total RNA was examined by 1% agarose gel electrophoresis, the quantity was determined based on the absorbance at 260 nm (A_260_), and the purity was analyzed based on the absorbance ratio at 260 and 280 nm. For pure RNA the ratio of A260/280 is ~2.0(A_260/280_; Amersham Biosciences GeneQuant, Pittsburgh, PA, USA). cDNA was synthesized from total RNA (1 μg) using AMV reverse transcriptase XL (Takara Bio, Inc., Shiga, Japan). β-actin served as an internal control. To create real-time PCR standards, TGFα and β-actin were amplified using reverse transcriptase-PCR and specific primers. The amplicons were cloned into the pMD18-T vector (Takara) and the sequence was confirmed by sequencing. The purified recombinant DNA was quantified and then serially diluted in ultra-pure water to a final concentration ranging from 10^7 ^to 10^1 ^copies/μL. For quantification standards, we used 1-μL aliquots of 10-fold serial dilutions of plasmid DNA. A new standard curve was run for each real-time PCR, and each test run included a control containing non-target DNA. The amount of TGFα mRNA was normalized to the expression values of the housekeeping gene β-actin.

### TGFα enzyme-linked immunosorbent assay (ELISA)

Medium was harvested from cells grown on 6-well dishes. Samples were processed using the human TGFα quantikine ELISA kit (R&D systems, Inc., Minneapolis, MN, USA) according to the manufacturer's instructions.

### Statistics

Results are presented as the mean ± standard error (SE) of at least three experiments. Statistical comparisons of sequence-dependent effects were conducted by student's *t *tests. Statistics and graphs were generated using the GraphPad Prism software (ver. 5.00 for windows, GraphPad Software Inc, CA, USA).

## Results

### The potential mechanisms for gefitinib resistance in PC-9/GR cells

**Figure 1:**
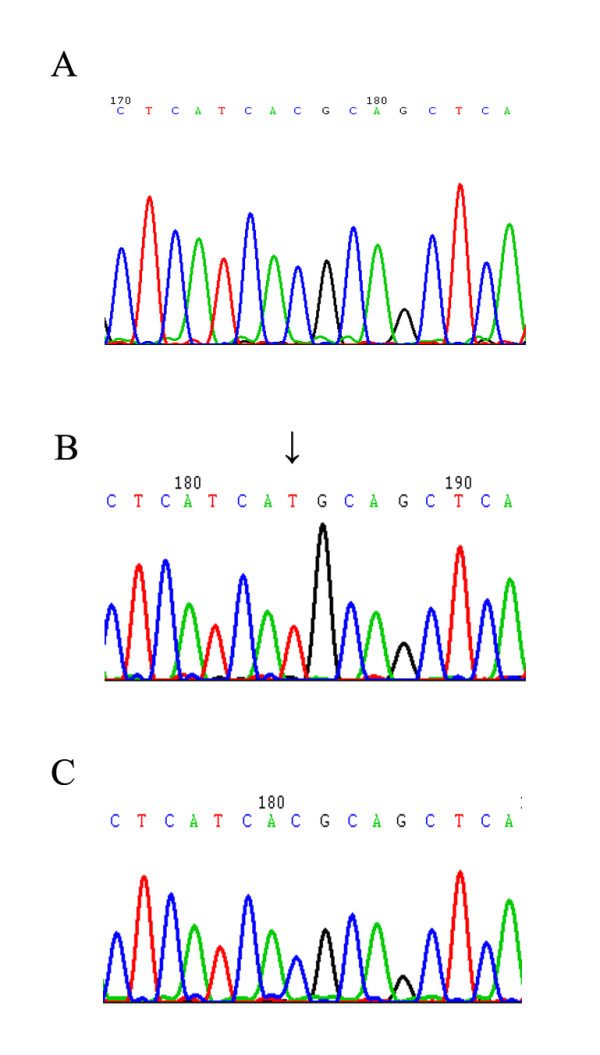
**PC-9/GR exon 20 sequence**. A. Wild type by direct sequencing. B. 3 of 20 clones had ACG > ATG T790M mutation by clone sequencing. C. 17 of 20 clones were wild type

The gefitinib-acquired resistant PC-9/GR cells were obtained 6 months after initial exposure. These cells were 100-fold more resistant than parental cells(Table-1). Additional mutations were not detected by direct sequencing. The PCR product of PC-9 and PC-9/GR were subcloned into a plasmid vector, then the inserts were randomly selected and sequenced. Among the 20 sequences from PC-9/GR, we found 6 sequences were wild-type, 11 had only the 15-bp deletion, and 3 had both the 15-bp deletion and T790M mutation. These T790M mutation seemed to be the cause of gefitinib resistance. Of 10 colones isolated from PC-9, 4 had no mutation, and the others had the 15-bp deletion (Figure 1).

### Sequence-dependent antiproliferative effects differed between EGFR-TKI-sensitive and EGFR-TKI-resistant NSCLC cell lines

**Figure 2:**
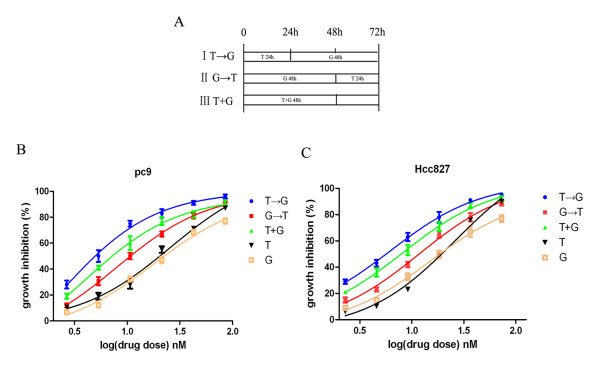
**The antiproliferative effect with paclitaxel and gefitinib is sequence-dependent**. A. Schema of sequential treatment. B and C. The sequence of paclitaxel followed by gefitinib produced the most potent antiproliferative effect in PC-9 and Hcc827 cells. T → G, paclitaxel followed by gefitinib. G → T, gefitinib followed by paclitaxel. T + G, concomitant paclitaxel and gefitinib. T, paclitaxel alone for 72 h. G, gefitinib alone for 72 h.

To evaluate the antiproliferative effects of paclitaxel and gefitinib treatment, we performed a series of MTT cell growth assays. Treatment with gefitinib alone for 72 h resulted in a dose-dependent inhibition of cancer cell growth. Table 1 summarizes the IC_50 _of these two drugs. PC-9 and Hcc827 were highly sensitive to gefitinib, while H1650 exhibited primary resistance and PC-9/GR showed acquired resistance to gefitinib. All of these cell lines demonstrated similar sensitivities to paclitaxel. We evaluated the growth inhibition effect on Hcc827, PC-9, PC-9/GR, and H1650 of three different sequences of combined paclitaxel and gefitinib treatments. Figure 2a shows the schema for *in vitro *sequential treatment between paclitaxel and gefitinib. As shown in Figure 2b and 2c, the antiproliferative effects between paclitaxel and gefitinib were sequence-dependent. Although the differences were not marked, the sequence of paclitaxel followed by gefitinib was most efficacious against EGFR-mutant NSCLC cells.

**Table 1:** IC50 values for each drug were calculated by performing dose response experiments with gefitinib and paclitaxel

IC50	Hcc827	PC-9	PC-9/GR	H1650
Paclitaxel	2.27 ± 0.15nM	2.33 ± 0.47nM	2.59 ± 0.62nM	3.29 ± 0.35nM

Gefitinib	16 ± 1.12nM	19 ± 2nM	2.1 ± 0.39 μM	9.37 ± 0.52 μM

**Figure 3:**
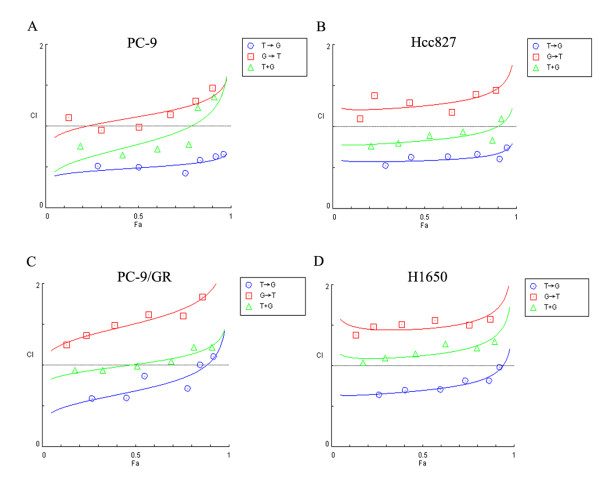
**Synergism of sequence-dependent cytotoxicity between paclitaxel and gefitinib**. Points, mean values of three different experiments. ○ T → G, paclitaxel followed by gefitinib. □ G → T, gefitinib followed by paclitaxel. △ T + G, concomitant paclitaxel and gefitinib. The sequence T → G produced the most potent cytotoxic growth inhibition. The T → G sequence resulted in a synergistic effect.

The calculation of CI values revealed that the sequence of paclitaxel followed by gefitinib produced synergistic effects. In Hcc827 and PC-9 cell lines, which were highly sensitive to EGFR-TKIs, the sequence of paclitaxel followed by gefitinib resulted in synergistic effects, with mean CI values of 0.63 in Hcc827, 0.54 in PC-9. Concomitant administration of the drugs resulted in both synergistic and additive effects, with mean CI values of 0.88 in Hcc827, 0.91 in PC-9. The gefitinib followed by paclitaxel sequence resulted in an additive and antagonistic interaction with mean CI values of 1.29 in Hcc827, 1.16 in PC-9 (Figure 3a,b). In the PC-9/GR cell line, which exhibited acquired resistance to EGFR-TKIs, the sequence of paclitaxel followed by gefitinib resulted in a synergistic and additive interaction with increasing drug concentrations (mean CI, 0.81). In contrast, the sequence of gefitinib followed by paclitaxel resulted in an antagonistic effect (mean CI, 1.52). Concomitant treatment with the two drugs resulted in additive and antagonistic effects (mean CI, 1.05)(Figure 3c). In the H1650 cell line, a synergistic and additive growth inhibition effect was found with the sequence of paclitaxel followed by gefitinib (mean CI, 0.77). In contrast, the sequence of gefitinib followed by paclitaxel resulted in an antagonistic interaction (mean CI, 1.5). Simultaneous administration of the two drugs resulted in an additive and antagonistic interaction (mean CI, 1.18)(Figure 3d). Each experiment was repeated in triplicate.

### Sequence-dependent modulation of the EGFR signaling pathway

**Figure 4:**
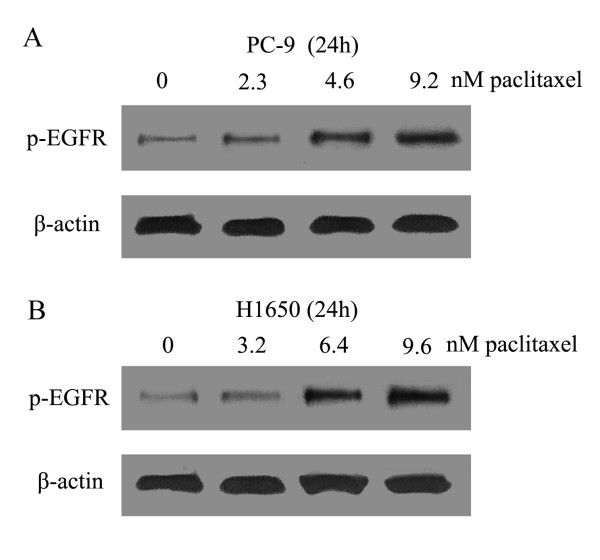
**Effect of paclitaxel on EGFR phosphorylation**. PC-9 and H1650 cells were exposed to 1, 2 and 3 fold IC50 dose paclitaxel for 24 h. A dose-dependent increase in EGFR phosphorylation levels were observed.

**Figure 5:**
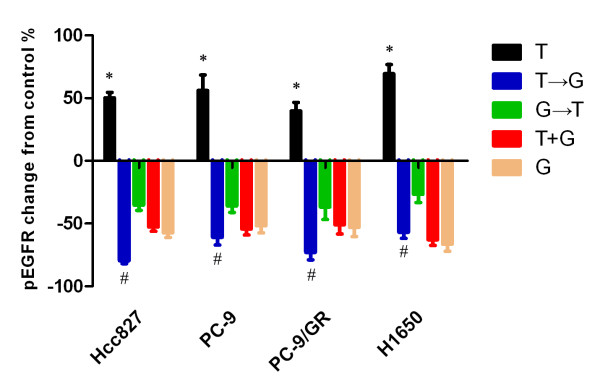
**Sequential modulation of EGFR phosphorylation**. Cells were exposed to the IC50 dose of gefitinib and paclitaxel using different sequences. *Paclitaxel significantly activated the EGFR pathway compared with control after paclitaxel exposure for 24 h (p < 0.05). # p < 0.05. The phosphorylated EGFR (p-EGFR) level after treatment with paclitaxel followed by gefitinib was lower than that for gefitinib followed by paclitaxel.

To gain insight into the mechanisms involved regulating the interaction between paclitaxel and gefitinib, we examed the effect of paclitaxel on EGFR phosphorylation. In cell lines of PC-9 and H1650, a dose-dependent increase in pEGFR levels were observed 24 hous after 1, 2 and 3 fold IC50 dose of paclitaxel exposure(Figure 4). Because EGFR-TKIs specifically target the tyrosine kinase activity of EGFR, we analyzed the sequence-dependent modulation of the EGFR signaling pathway using an IC_50 _dose of paclitaxel plus gefitinib combination. As shown in Figure 5, gefitinib significantly inhibited EGFR phosphorylation (*p *< 0.01). When Hcc827, PC-9, PC-9/GR and H1650 cell lines were exposed to paclitaxel alone, phosphorylated EGFR (pEGFR) levels increased significantly after paclitaxel exposure for 24 h than that in untreated control (mean difference, +50% in Hcc827, +56% in PC-9, +39% in PC-9/GR and +69% in H1650 cells respectively, *p *< 0.05). The increase in pEGFR induced by paclitaxel was blocked by adding gefitinib. In the reverse sequence, gefitinib treatment for 48 h significantly inhibited pEGFR levels, compared with the untreated control level of pEGFR, and subsequent paclitaxel exposure for 24 h enhanced pEGFR levels. The paclitaxel followed by gefitinib sequence produced significantly greater pEGFR inhibition than did the reverse sequence (mean difference, -58% in Hcc827, -38% in PC-9, -35% in PC-9/GR and -30% in H1650 respectively, *p *< 0.05).

These data suggest that paclitaxel may also have a self-limiting effect on its toxicity, via activation of the EGFR tyrosine kinase phosphorylation, leading to increased proliferation and survival of paclitaxel-mediated cytotoxicity. The increase in pEGFR can be blocked by adding gefitinib, which produces a synergistic effect. In the reverse sequence, however, the sequence of gefitinib followed by paclitaxel interferes with the inhibition of pEGFR by gefitinib.

### Paclitaxel-induced primary activation of EGFR is blocked by a neutralizing antibody to TGFα

**Figure 6:**
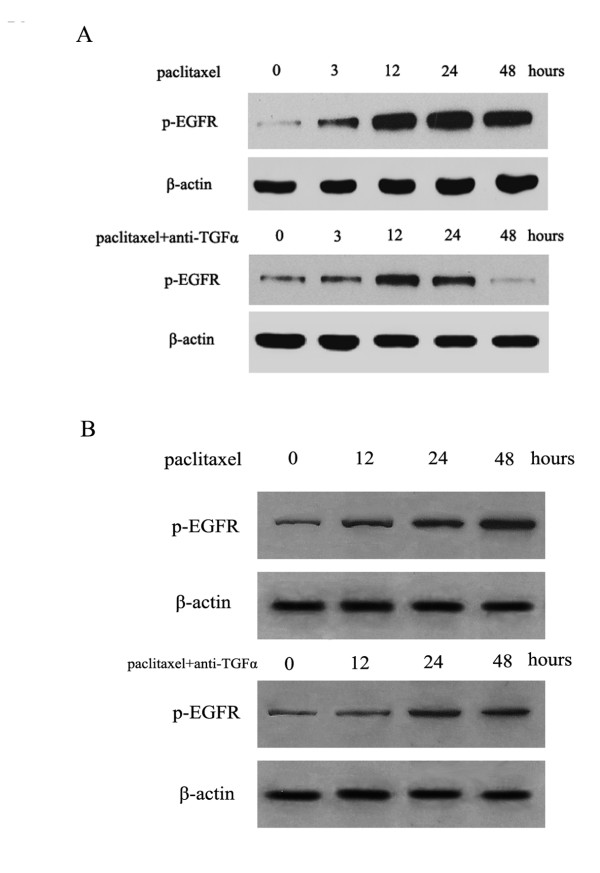
**Paclitaxel-induced activation of EGFR is blocked by adding neutralizing TGFα antibody**. The dose of paclitaxel is 2-fold IC50. The dose of TGFα antibody is 0.15 μg/mL A.PC-9 B. H1650

Several studies have demonstrated that chemotherapy activates EGFR phosphorylation [24]. Multiple mechanisms allow for EGFR activation and persistent tumor cell proliferation, including activating mutations, receptor amplification, and ligand up-regulation [25,26]. Other studies have found that radiation causes release of TGFα, which is responsible for activation of the EGFR signaling pathway [13]. We next examined whether activation of the EGFR signaling pathway induced by paclitaxel is dependent on TGFα. Paclitaxel caused EGFR activation in PC-9 and H1650 cells after a 12-h exposure and lasted for 48 h. Addition of a neutralizing antibody to TGFα reduced the activation of EGFR (Figure 6). These data demonstrated that paclitaxel-induced EGFR activation was dependent on the function of TGFα.

**Figure 7:**
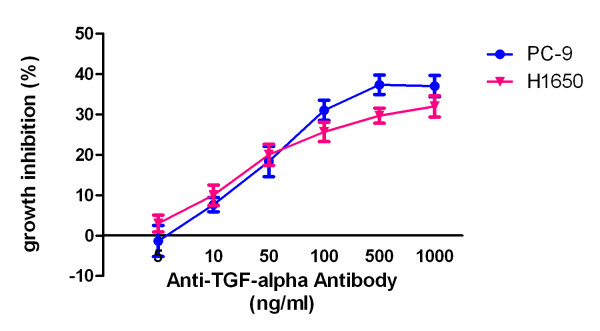
**Growth inhibition of PC-9 and H1650 by an anti-TGFα.antibody.** PC-9 and H1650 cells were grow into a 96 well plate. The following day, the cells were incubated with indicated concentration of anti-TGFα antibody for 72 h. The inhibition ratio were determined by MTT assay.

TGFα is a cognate ligand for EGFR, mediate tyrosine phosphorylation of the receptor. We then determine the growth inhibition effect of an anti-TGFα antibody. PC-9 and H1650 cells were grow into the medium containing increasing concentration of anti-TGFα antibody for 72 hours. The antibody show a modest growth inhibition effect, inhibited the growth of PC-9 and H1650 by about 30% at 500 ng/ml (Figure 7).

### Sequence-dependent TGFα expression and release are responsible for the sequence-dependent EGFR pathway modulation

**Figure 8:**
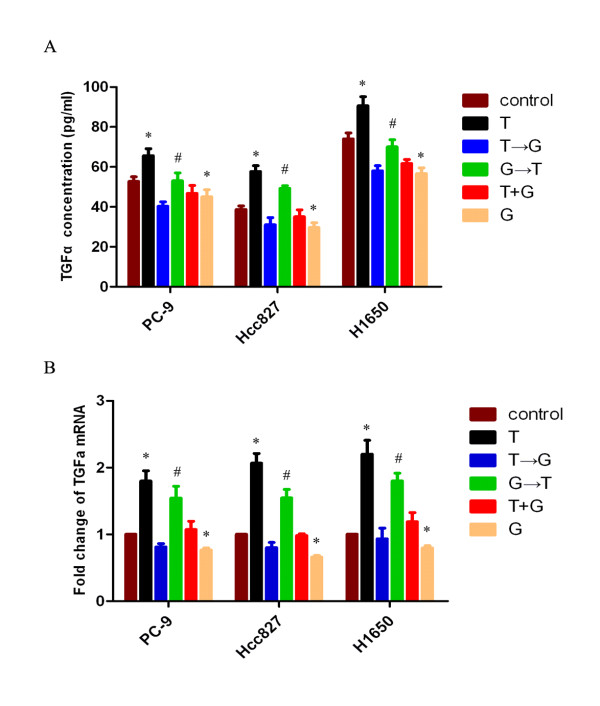
**Sequential modulation of TGFα release and expression**. Cells were exposed to IC50 dose of paclitaxel and gefitinib with different sequence. A, standard enzyme-linked immunosorbent assay analysis of soluble TGFα levels in PC-9, Hcc827 and H1650 cells. *p < 0.05, significantly different from control. #P < 0.05, T→G VS G→T. B, reverse transcription-polymerase chain reaction analysis of TGFα expression. *p < 0.05, significantly different from control. #P < 0.05, T→G vs. G→T.

To further evaluate whether sequential modulation of the EGFR pathway is due to the release of TGFα, PC-9, Hcc827 and H1650 cells were treated with sequential paclitaxel and gefitinib as indicated in the treatment schema of Figure 2a. Medium was subjected to ELISA to verify soluble TGFα levels and was normalized to cell numbers. As shown in Figure 8a, paclitaxel exposure for 24 h significantly stimulated TGFα release. Gefitinib significantly inhibited the release of TGFα (*p *< 0.05). The paclitaxel-followed-by-gefitinib sequence resulted in a significant reduction of TGFα levels, compared with the reverse sequence (*p *< 0.05).

To gain further insight into the mechanisms involved in regulating the interaction between paclitaxel and gefitinib, we examined changes in TGFα mRNA expression in treated PC-9, Hcc827 and H1650 cells. Quantitative PCR analysis of TGFα mRNA expression levels showed that in comparison with the respective control, paclitaxel significantly increased (*p *< 0.01), while gefitinib significantly decreased TGFα mRNA expression (*p *< 0.05). The paclitaxel-followed-by-gefitinib sequence significantly decreased TGFα mRNA expression, compared with the reverse sequence (Figure 8b; *p *< 0.05).

## Discussion

The present study demonstrated that the antiproliferative effect with paclitaxel and gefitinib is sequence-dependent in EGFR-mutant NSCLC cells. We found that the sequence of paclitaxel followed by gefitinib produced the most efficacious antiproliferative effect. Other studies have demonstrated sequence-dependent interaction between EGFR-TKIs and chemotherapy in human cancer cell lines [27]. These studies also revealed that pretreatment with erlotinib caused G1 arrest and effectively abrogated the activity of chemotherapy, resulting in decreased cytotoxicity and decreased apoptosis [28-30]. Solit *et al*. reported schedule-dependent efficacy in an NSCLC xenograft model with paclitaxel and gefitinib [31]. Our study was novel because we found that sequence-dependent modulation of the EGFR signaling pathway might play a role in the sequence-dependent interaction in NSCLC cells harboring an EGFR mutation. We found that paclitaxel-induced activation of EGFR was dependent on the function of TGFα. Additionally, TGFα expression and release were sequence-dependent, which may responsible for the sequence-dependent interaction between paclitaxel and gefitinib.

As EGFR-TKIs gefitinib and erlotinib are active in patients with NSCLC harboring EGFR mutations, combining them in treatment with chemotherapy was deemed promising [8,9]. However, in the INTACT-1, INTACT-2, TALENT, and TRIBUTE clinical trials, the addition of gefitinib or erlotinib to first-line chemotherapy failed to improve survival [32-35]. In a phase II trial of CALGB 30406, the concurrent combination of paclitaxel plus carboplatin with erlotinib showed a similar efficacy compared with erlotinib alone in patients with advanced lung adenocarcinoma [36]. Gandara *et al*. [37,38] proposed two hypotheses that seem reasonable to explain the negative results. First, patients were not selected based on a predictive response marker, although other studies have reported that activating mutations in the EGFR tyrosine kinase domain were associated with a dramatic response to gefitinib and erlotinib. Second, potentiation of the antagonistic interaction between concurrent EGFR-TKI and chemotherapy may have played a role, because *in vitro *and *in vivo *studies of administration of concurrent EGFR-TKI and chemotherapy have suggested antagonism. Davies *et al*. [39] have proposed the pharmacodynamic separation model: EGFR-TKIs primarily cause cell cycle arrest and accumulation of cells in G1; and thus, when administered concurrently with chemotherapy, can interfere with cell cycle-specific cytotoxicity. Our previous study[12] and other study[30] found that EGFR-TKI induces G1 arrest, which produced a negative interaction when combined concurrently with chemotherapy or followed by chemotherapy.

Chemotherapy has been shown to activate multiple signaling pathways [40] and, recently, to activate the EGFR pathway as well as to enhance ubiquitination and degradation [41,42]. The intracellular TK activity of EGFR is increased as a consequence of binding of various cognate ligands, including EGF, TGFα, and amphiregulin, leading to homodimerization of two EGFRs or heterodimerization of EGFR with other family members [43]. Improper activation of EGFR TK results in increased malignant cell survival, proliferation, invasion, and metastasis [44,45]. Several chemotherapy drugs were found to activate the EGFR signaling pathway [41]. Radiation has also been demonstrated to activate the EGFR signaling pathway by causing the release of TGFα, which is responsible for secondary activation of EGFR, mitogen-activated protein kinase (MAPK), and Janus kinase (JNK) pathways [13]. In the present study, neutralizing TGFα abolished the paclitaxel induced-activation of EGFR, and paclitaxel may stimulate release of TGFα to activate the EGFR pathway.

Autocrine loops are established when soluble factors secreted by cells bind to and stimulate receptors on their own surfaces [46]. TGFα is a cognate ligand for EGFR and can establish an autocrine loop that leads to receptor hyperactivity[47]. Ciardiello, et al. found that a TGFα-EGFR autocrine growth pathway is active in cancer cell lines, and gefitinib produced a dose-dependent inhibition of the secretion of TGFα[48]. Our data suggest that paclitaxel may exert a self-limiting effect on its ability to kill and reduce the proliferation of autocrine-regulated tumor cells, potentially by increasing both the rates of transcription and activation of TGFα. Increased expression and release of TGFα leads to both increased EGFR and activation of the downstream signaling pathway, which, in turn, leads to both increased proliferation of tumor cells as well as other enhanced cytoprotective responses. Increased TGFα expression and release can be blocked by subsequent administration of gefitinib, which can block TGFα-triggered EGFR signaling pathway activation and produce a synergistic effect. In contrast, initial exposure to gefitinib decreases TGFα expression and release, and subsequent paclitaxel treatment enhances TGFα expression and release, producing an antagonistic effect.

Cytotoxic chemotherapy treatment for patients with advanced NSCLC has reached a plateau, but further improvements are expected with the integration of targeted therapies[49,50]. EGFR-TKIs treatment concurrent with chemotherapy failed to improve survival. Alternative schedules of TKI administration in combination with chemotherapy, such as sequential and pulse administration, are under investigation. In the Fast-Act trial, a randomized phase II trial of sequential erlotinib and chemotherapy as first-line treatment of patients with advanced NSCLC, gemcitabine plus cisplatin or carboplatin (GC) were sequentially followed by erlotinib (GC-E) versus GC followed by a placebo (GC-P). The results revealed that GC-E significantly improved PFS compared with GC-P [51]. Based on these results, Fast-Act II, a randomized phase III trial of sequential erlotinib and chemotherapy, is underway[52].

In the final analysis of the pivotal IPASS trial, although there is significant improvement in PFS and RR for patients receiving gefitinib, overall survival was similar for gefitinib and carboplatin/paclitaxel, reported Chin-Hsin Yang[53]. The effect of the initial randomized treatment therapy on overall survival is likely to have been confounded by subsequent study, particulary, the switching of patients to the alternative study treatment. This suggests the sequence of therapy may not make a significant impact in patients with mutant EGFR, as long as such patients are exposed to an EGFR TKI in general, and whether it is given first-line or later translates into the same overall survival benefit.

The limitation of this study is that because the short exposure time, the interaction of sequential treatment in vitro may not correlate the interaction between first-line and second-line treatment in clinic. The drug interaction patterns observed in vitro may not be similar to those observed clinically. Because the culture condition can perturb cell function and potentiate drug sensitivity, obtaining good correlations can be problematic. Pharmacokinetics, pharmacodynamics, rescue effects and toxicity to normal cells/tissues that affect drug action in clinical setting are not considered by in vitro assays. Because each patient has a unique pharmacogenetic makeup, correlating in vitro and clinical results is often not a straightforward process. However, our study provided a rational for the ongoing clinical investigation of sequential treatment of NSCLC.

## Conclusions

In summary, our studies have demonstrated that sequential administrations of paclitaxel followed by gefitinib result in the most effective cytotoxic effects in NSCLC cell lines harboring EGFR mutations. The sequence-dependent modulation of TGFα expression and release might be responsible for the synergistic interaction between paclitaxel and gefitinib. Although the extrapolation of in vitro data to the clinical setting should be considered with caution, these results may have implications for the rational development of chemotherapeutic regimens for the treatment of NSCLC.

## Competing interests

Yi-long Wu has received speaking honoraria from AstraZeneca, Eli lilly, F.Hoffmann-La Roche and Pfizer. No other potential conflict of interest relevant to this article was reported.

## Authors' contributions

HC designed thses experiments, performed part of these experiments and drafted this manuscript. YW designed these experiments, analyzed the results, guided these experiments and revised this manuscript. SA and ZC carried out RT-PCR. DS and YZ carried out the western blot, XZ analyzed the experiment results. JS carried out part of the MTT assays. All authors have read and approved the final manuscript.
